# Improvements to water purification and sanitation infrastructure may reduce the diarrheal burden in a marginalized and flood prone population in remote Nicaragua

**DOI:** 10.1186/1472-698X-10-30

**Published:** 2010-12-08

**Authors:** Sheri A Denslow, Jess Edwards, Jennifer Horney, Rodolfo Peña, Daniel Wurzelmann, Douglas Morgan

**Affiliations:** 1 Department of Epidemiology, The Gillings School of Global Public Health, University of North Carolina, Chapel Hill, NC, USA; 2 Centro de Investigación en Demografía y Salud (CIDS), UNAN-León, Nicaragua; 3 Department of Gastroenterology and Hepatology, University of North Carolina School of Medicine, Chapel Hill, North Carolina, USA

## Abstract

**Background:**

The isolated northern region of Nicaragua has one of the highest rates of diarrheal disease in Central America. Political and environmental hardships faced by inhabitants of this region are contributing factors to this health inequity. The aim of this study was to assess the relationship between water and latrine infrastructure and the prevalence of diarrhea in this region.

**Methods:**

A population-based, cross-sectional survey of women of reproductive age was conducted in the Sahsa region of northern Nicaragua in July, 2009. Households were selected by two stage cluster sampling methodology. A questionnaire was administered in Spanish and Miskito with assessment of household and socioeconomic conditions, sanitation practices, and health care access. Diarrhea prevalence differences at the household level over a two week reporting period were estimated with a standardized instrument which included assessment of water treatment and latrine use and maintenance.

**Results:**

There were 189 women enrolled in the current study. The use of water purification methods, such as chlorine and filters, and latrine ownership were not associated with reduced prevalence of household diarrhea in the two week reporting period. Latrine overflow, however, was associated with an increased prevalence of diarrhea during the same two week period [adjusted prevalence difference and 95% CI: 0.19 (0.03, 0.36)].

**Conclusions:**

Simple, low cost interventions that improve water and latrine infrastructure may reduce the prevalence of diarrheal disease in the isolated regions of Nicaragua and Central America.

## Background

Diarrhea remains a major health burden in resource limited nations, contributing substantially to morbidity and mortality. It is estimated that worldwide diarrheal disease leads to 1.87 million deaths each year in children under five, accounting for 19% of the total deaths in this age group[1]. Nearly 80% of these deaths occur in developing nations. Diarrheal illness is common in Nicaragua, particularly in the North Atlantic Autonomous Region (Región Autónoma del Atlántico Norte, RAAN) province, where household sanitation measures are not in common use. The Nicaraguan Ministry of Health (MINSA) has found that the RAAN has one of the highest incidences of diarrheal disease in Nicaragua. Mortality due to diarrhea accounts for 7.3% of deaths per year in the RAAN, compared to 1 - 2% of deaths in other regions of Nicaragua[2].

The RAAN is an extensive remote region in northern Caribbean Nicaragua, with a history of geographic and political isolation. One unpaved road connects the RAAN with the populated Pacific coastal areas of the country. The inland Tasba Pri region, with the central municipality of Sahsa, is located approximately 100 km from the Atlantic coast and 400 km east of the capital Managua. While Nicaragua as a whole is primarily Hispanic Mestizo, the RAAN population is ethnically and culturally diverse, with multiple indigenous groups, including Miskito, Mayagna, and Creole. Many populations relocated to the Sahsa region as refugees from the "crossfire" from the conflicts of the Sandinista Revolution and Contra war of the 1970 s and 1980s[3]. Many communities in the municipality of Sahsa are accessible only by canoe or footpath, and are without communication. The primarily agricultural region alternates between the harsh realities of dry months with little water available and the rainy season where an overabundance of water leads to flooding and muddy conditions. Due to the political reality, the geographic isolation of the region, and the distinct ethnic groups, the inhabitants of Sahsa have been marginalized, with significant health inequities.

The RAAN was further affected by Hurricane Felix, a Category 5 hurricane, which struck the Atlantic coast of Nicaragua in September, 2007. Hurricane Felix accentuated the burden of diseases in the region, and in particular diarrheal diseases. In response, investigators from the University of North Carolina at Chapel Hill (UNC) and the Universidad Autónoma de Nicaragua, León (UNAN) have partnered (Collaborative Sahsa Health Initiative, CSHI) to identify the regional health needs, with the future goal of specific interventions. This builds upon the existing UNAN surveillance systems of western Nicaragua[4].

The aim of this study was to estimate the prevalence of diarrheal disease in the Sahsa region, and identify associations with reported water sanitation and hygiene infrastructure in this region of Nicaragua. Specifically, this study addresses the impact of modifiable exposures (water sanitation and waste management) on the prevalence of diarrheal disease.

## Methods

### Study design

A two stage cluster sampling method (known as 30/7)[5] was used for random population-based selection of households from the 54 communities of the Sahsa region. In the first stage, 30 communities (primary sampling units) were selected with probability of selection proportionate to population size with replacement[5,6]. Three of the thirty selected primary sampling units were excluded in the field due to impossible travel conditions (2 units) and political instability (1 unit), resulting in a sampled population of 27 primary sampling units in 16 communities. In the second stage, 7 households were selected from each of the primary sampling units. Because satellite imagery and detailed maps were unavailable in this region, households within each cluster were randomly selected for interview using a "compass" method undertaken by the interview team on site in the community. In this method, starting at the most centralized school or church, a pen was spun to determine the direction. Surveyors would walk in that direction, down roads and across fields, until the first residence was reached. After each survey, again a pen was spun to determine the direction of travel to find the next household.

### Study instruments

Women were interviewed in July of 2009 about household characteristics, sanitation practices, diarrhea, family planning, and health care access. Interviews were conducted in Spanish or Miskito, depending on the language of the woman. Social promoters supported the interviews in Miskito. Study participants were questioned about the prevalence of diarrhea in the household in the past two weeks using the standard CDC instrument. For this study, diarrhea was coded as a dichotomous variable and was defined as 3 or more loose or watery stools or any stool with blood in a 24-hour period. Two week prevalence of diarrhea was assessed at the household level, as many of the risk factors examined in this analysis occur at the level of the household, as opposed to the level of the individual. The study protocol was approved by the Institutional Review Boards at the University of North Carolina, Chapel Hill and the Universidad Nacional Autónoma de Nicaragua, León. Participants gave verbal consent prior to enrollment in the study and collection of data.

Water and sanitation infrastructure was measured through a series of questions about drinking water source, water treatment, and latrine use. Respondents indicated where they obtained their water (indoor or communal piping, private or communal well, river or creek, natural spring, other) and how they treated it prior to drinking (no treatment, chlorine, filter, other). Latrine use was assessed through two questions that ascertained if the household used a latrine (no, yes) and if so, how many times the latrine overflowed in the past year. Latrine use was recoded as a nominal categorical variable to combine these attributes (no latrine, latrine with no overflow, latrine with at least one reported overflow).

The United Nations standardized Poverty Index, validated in Nicaragua[4], was used to assess socioeconomic status and living conditions. This included measures of household structure, access to potable water, sanitation, number of individuals in the household, education level, and employment. The presence of *tambos*, stilts that support a house, was recorded as the proxy for houses built in a known flood zone. The remoteness of each household was estimated by the reported travel time to the nearest health center (continuous variable, recorded in minutes).

### Statistical analysis

Prevalence differences (PD) and 95% confidence intervals were estimated to examine associations between diarrhea prevalence with water treatment and latrine use. Covariates were assessed for effect measure modification by comparing stratified PD estimates and testing for heterogeneity using the Wald Heterogeneity test p-value with an a priori criterion of 0.20. Potential confounders were identified using directed acyclic graphs (DAGs) informed by recent literature on the subject[7,8]. Potential confounders were assessed for their inclusion in the linear regression models through backwards elimination, where variables were removed from the full models one at a time in the order of their change-in-estimate from the singly adjusted prevalence differences. If elimination of the variable resulted in a change in the PD estimate generated by the full model of greater than 0.02, the variable was retained in the model[9]. Data were analyzed in SAS 9.2 (SAS Institute Inc., Cary, NC).

## Results

Surveyors from UNC and UNAN accompanied by a local health promoter conducted 189 interviews in the selected 27 primary sampling units comprising 16 communities in the Sahsa region in July 2009. Of those eligible to participate, 94.5% completed the survey (189/200). Nine women refused the interview, while one interview could not be conducted due to language barriers, and one interview was incomplete.

Study participants were predominantly of Mestizo ethnicity (Table 1). The majority (n = 145, 79%) of respondents indicated that the head of household had not received education beyond primary school. Nearly half (n = 77, 41%) of the households were in flood zones, as estimated by the presence of *tambos *(stilts). Half (n = 85, 47%) of the study participants lived in remote areas greater than one hour away from the nearest health center. Households interviewed during the study obtained water from piping (n = 78, 41%), wells (n = 60, 32%), rivers (n = 30, 16%) and springs (n = 17, 9%) and the majority (n = 103, 57%) of families used no method of water purification. The households that did employ a method of water purification used chlorine or water filters. The majority of households (n = 146, 78%) used a latrine, and of those families, 51 (37%) experienced an overflow of the latrine in the previous year.

**Table 1:** Characteristics of Study participants in the Collaborative Sahsa Health Initiative in the North Atlantic Autonomous Region, Nicaragua, 2009 (N = 189).

		Number of households^A^	% of total households^B^
Race^C^			
	Mestizo	171	91
	Miskito	16	9
	Other	1	1
Education^D^			
	Illiterate	33	18
	Literate, No education	31	17
	Preschool	1	1
	Primary School	80	43
	Secondary School	28	15
	University	3	2
	Trade school	1	1
	Don't Know	3	2
	No Response	5	3
Tambos^E^			
	Yes	77	41
	No	111	59
Time to health center^F^			
	Under 1 hour	96	53
	1 hr - <2 hrs	29	16
	2 hrs - <3 hrs	21	12
	3 hrs - <4 hrs	16	9
	4 hrs or more	19	11
Water Source			
	Piping	78	41
	Well	60	32
	River	30	16
	Natural spring	17	9
	Other	4	2
Water purification method			
	None	103	57
	Chlorine	54	30
	Filter	25	14
	Missing	1	
Use latrine			
	Yes	146	78
	No	41	22
	Missing	2	
Latrine overflow in past year			
	Yes	51	37
	No	88	63
	Missing	7	
Diarrhea	Yes	77	41
	No	112	59

^A ^Number of households surveyed within the community.

^B ^Percentage of total surveyed households (N = 189).

^C^Race of the head-of-household

^D ^Education level of the head-of-household

^E ^House on stilts, indicative of location in a flood-prone region.

^F ^Self-reported time to travel to the nearest health center.

In the study sample, 41% (n = 77) of households reported an instance of diarrhea in the past two weeks. The two week household prevalence of diarrhea was lower in households using water purification, such as chlorine and filters (N = 29, 37%), than for households drinking untreated water (N = 40, 41%) (Table 2). Families drinking from wells and rivers had a prevalence of diarrhea in the previous two weeks 10% and 16% higher, respectively, than those drinking from piping, though both 95% confidence intervals (CI) encompassed the null. Households reporting that they did not have a latrine were more likely to report diarrhea in the previous two weeks. Forty-nine percent of households without a latrine reported at least one case of diarrhea within the past two weeks, whereas only 38% of households with latrines reported diarrhea (Table 2).

**Table 2:** Water and sanitation infrastructure and household prevalence of diarrhea in the North Atlantic Autonomous Region, Nicaragua, 2009 (N = 189).

	Diarrhea^A^	No Diarrhea	PD (95% CI)
Water Source			
Piping	26	52	0
Well	26	34	0.10 (-0.06, 0.26)
River	15	15	0.17 (-0.04, 0.37)
Natural spring	8	9	0.14 (-0.12, 0.40)
Missing	2	2	
Water Treatment			
None	44	61	0
Chlorine	20	34	-0.05 (-0.21, 0.11)
Filter	9	16	-0.06 (-0.27, 0.15)
Missing	4	1	
Latrine Use^B^			
No	20	21	0
Yes	56	90	-0.10 (-0.28, 0.07)
Missing	1	1	
Latrine overflow^C^			
No	28	60	0
Yes	26	25	0.19 (0.02, 0.36)
Missing	2	5	

PD = prevalence difference, PR = prevalence ratio

^A ^Two week prevalence of household diarrhea

^B ^Household latrine

*^C ^Within household that had a latrine, did that latrine overflow (1 or more times) in the past year*.

Five potential confounders of the relationship between water treatment method and diarrheal disease were assessed: water source, residence in a flood zone, socioeconomic status (head of household education level), number of individuals in the household, and time required to travel to the health center (an indicator of remoteness). In the linear regression model, after backward elimination, only water source remained in the model. The adjusted PD and 95% CI comparing two-week prevalence of diarrhea among households who treated water with filters to households who drank untreated water was -0.12 (-0.33, 0.10) (Table 3). The PD for chlorine was -0.05 (-0.21, 0.11).

**Table 3:** Prevalence differences for the relationship between water treatment method and diarrheal disease^A^ in the North Atlantic Autonomous Region, Nicaragua, 2009.

	**Final Model**^**B**^	**Full Multivariate Model**^**C**^
	PD (95% CI)	PD (95% CI)
Water Treatment		
None	0	0
Chlorine	-0.05 (-0.21, 0.11)	-0.07 (-0.24, 0.09)
Filter	-0.12 (-0.33, 0.10)	-0.13 (-0.36, 0.10)

PD = prevalence difference

^A ^Two week prevalence of household diarrhea

^B^The final model was adjusted for water source only.

^C^The full multivariate model was adjusted for water source, tambos (as a proxy for location in a flood prone region), time to the health center, number of individuals in the household, and education level of the head of household (as a proxy for socioeconomic status)

For assessment of the relationship between latrine use, latrine overflow, and diarrhea, three potential covariates were considered: living in a flood zone, number of individuals in the household, and socioeconomic status (head of household education level). After backwards elimination, none of these covariates remained in the final model. The analysis shows that latrine overflow was associated with a higher prevalence of diarrhea (PD = 0.19 95% CI = 0.02, 0.36). There was some evidence of modification of the effect of having a latrine on the prevalence of diarrhea by latrine overflow. The PD comparing owning a latrine that did not overflow to not owning a latrine was -0.17 (-0.35, 0.01), while the PD comparing owning a latrine that did overflow to not owning a latrine was 0.02 (-0.18, 0.22) (Table 4).

**Table 4:** Linear regression models of the relationship between latrine use, latrine overflow, and diarrhea^A^ in the North Atlantic Autonomous Region, Nicaragua, 2009 (N = 189).

	**Final PD**^**B **^**(95% CI)**	**Full Multivariate Model**^**C**^ PD (95% CI)
Latrine Use^D^		
No Latrine	0	0
Latrine, no overflow	-0.17 (-0.35, 0.01)	-0.17 (-0.36, 0.01)
Latrine, overflowed^E^	0.02 (-0.18, 0.22)	0.03 (-0.18, 0.23)
Overflow		
No reported overflow	0	0
Reported overflow	0.19 (0.03, 0.36)	0.20 (0.03, 0.36)

PD = prevalence difference

^A ^Two week prevalence of household diarrhea

^B ^The final model was not adjusted for other covariates

^C ^The full multivariate models were adjusted for tambos (a proxy for living in a flood prone region), number of individuals in the household, and education level of the head of household (a proxy for socioeconomic status of the household)

^D ^Household latrine

^E ^Within household that had a latrine, did that latrine overflow (1 or more times) in the past year.

## Discussion

This population-based, cross-sectional study in the isolated Sahsa municipality of Nicaragua found a high prevalence of household diarrhea (41%), which is consistent with data from the Nicaragua Ministry of Health (MINSA)^2^. Importantly, the analysis suggests that latrine ownership with no reported overflow was associated with reduced household prevalence of diarrhea.

The use of prevalence differences in our analysis allows for a prediction of benefit from a change in exposure[10], with the number of needed interventions, or the number needed to treat (NNT) in order to reduce the household diarrheal burden by 1. Our data estimate that the NNT for water treatment with the use of filters is 17 households, and for chlorine treatment it is 20 homes. Similarly, the NNT is 10 for latrine use, and 6 for use of well-designed latrines which do not overflow. This suggests that an important reduction in diarrheal burden can be obtained with changes in the sanitation infrastructure.

Previous investigations addressing water source, water treatment, and latrine use have led to inconsistent conclusions on associations with diarrheal disease[11-17]. A recent review found that point-of-use chlorine treatment was associated with reduction in diarrhea in 9 of 10 reviewed studies[14]. In only 5 of these, however, was the association statistically significant. Sand filtration systems have been shown to be effective at reducing diarrhea [16,17]. Our results did identify an association of water source and treatment with reduced prevalence of diarrhea, but these findings were not statistically significant. For sanitation infrastructure, two studies have shown a reduction in diarrhea associated with latrine access[13,18]. Our results are similar in that we found a reduction in diarrhea for households with access to a latrine. These results, however, were not statistically significant. A statistically significant association was found for those with access to a properly situated latrine that does not overflow.

The discordant results may stem from the specific emphasis of the studies. While water purification and latrine use are factors which protect against diarrheal disease, few studies have addressed these factors in a remote, impoverished, and high prevalence region. This study suggests that proper latrine function is effective in preventing diarrhea even in areas with many other risk factors for diarrhea, by highlighting the importance of proper placement and maintenance of latrines. Ownership of a latrine that overflowed was associated with little to no change in the prevalence of household diarrhea compared to not owning a latrine (PD: 0.02, 95%CI: -0.18, 0.23), whereas ownership of a latrine that did not overflow was associated with an appreciable difference (PD: -0.17 95% CI: -0.36, 0.01). The identification of the potential causal interaction between latrine ownership and overflow may have important implications for directing future interventions. Without the interaction term, the PD model treats all households reporting latrines with equal risk, regardless of the proper functioning of the latrine, which may lead to inaccurate effect estimates.

The main strengths of this study include the population-based sampling of the isolated Sahsa region and the high subject response rate. The incorporation of local health promoters was a key factor in the high response rate. The assessment has several limitations. The study region is isolated and the travel required to conduct interviews in selected communities was challenging. Three initially selected communities could not be reached due to travel conditions and safety concerns. As detailed maps of the region were not available, second stage sampling used the "compass" approach for household selection. By allowing interview teams to select interview locations in this way, selection bias may have been introduced[6]. A further potential bias is that data are based on self-report. No inspections of the home were made to verify answers on water treatment or latrine set-up.

## Conclusions

This study provides a step forward in understanding the diarrheal disease burden in northern Nicaragua, which is potentially generalizable to other remote regions of Central America. It is suggested that low cost interventions to prevent latrine overflow may reduce the incidence of diarrheal disease, even in the presence of other risk factors. These findings provide support to the notion that public health interventions should be appropriate for the regional environment. This information may be helpful in directing international planning for infrastructure improvements aimed at addressing extreme poverty, such as those outlined in the United Nations millennium development goals adopted in 2000[19]. Simple, low cost interventions that improve water and latrine infrastructure may reduce the prevalence of diarrheal disease in the isolated regions of Nicaragua and Central America.

## Competing interests

The authors declare that they have no competing interests.

## Authors' contributions

SD and JE were involved in study design, helped with data collection, performed data analysis, and drafted the manuscript. JH participated in study design and helped to draft the manuscript. RP was involved in study conception and coordination. DW was involved in study conception and design and helped with data collection. DM was involved in study conception and coordination and was involved in critical revisions of the manuscript. All authors read and approved the final manuscript.

## Pre-publication history

The pre-publication history for this paper can be accessed here:

http://www.biomedcentral.com/1472-698X/10/30/prepub
